# Bottom-Up Coarse-Grained Modeling of DNA

**DOI:** 10.3389/fmolb.2021.645527

**Published:** 2021-03-17

**Authors:** Tiedong Sun, Vishal Minhas, Nikolay Korolev, Alexander Mirzoev, Alexander P. Lyubartsev, Lars Nordenskiöld

**Affiliations:** ^1^School of Biological Sciences, Nanyang Technological University, Singapore, Singapore; ^2^Department of Materials and Environmental Chemistry, Stockholm University, Stockholm, Sweden

**Keywords:** DNA condensation, coarse-grained model, molecular renormalization group, inverse Monte Carlo, multi-scale coarse-graining, force matching, relative entropy, persistence length

## Abstract

Recent advances in methodology enable effective coarse-grained modeling of deoxyribonucleic acid (DNA) based on underlying atomistic force field simulations. The so-called bottom-up coarse-graining practice separates fast and slow dynamic processes in molecular systems by averaging out fast degrees of freedom represented by the underlying fine-grained model. The resulting effective potential of interaction includes the contribution from fast degrees of freedom effectively in the form of potential of mean force. The pair-wise additive potential is usually adopted to construct the coarse-grained Hamiltonian for its efficiency in a computer simulation. In this review, we present a few well-developed bottom-up coarse-graining methods, discussing their application in modeling DNA properties such as DNA flexibility (persistence length), conformation, “melting,” and DNA condensation.

## 1. Introduction

Deoxyribonucleic acid (DNA) is the genetic information carrier of higher living organisms. To pass the encoded information from generation to generation, it has to allow efficient duplication and compaction. We still lack a full understanding of many fundamental aspects of DNA physics that determine DNA properties and function. To name a few, double-helical DNA encounters topological difficulties during replication (Postow et al., 2001) and compaction (Schiessel, 2003). Physically, curvature and elasticity of the DNA double helix are crucial for DNA compaction into chromatin and chromosomes, which is essential to cell division and gene expression regulation. Additionally, the conformational dynamics of DNA is crucial to its interaction with other macromolecules in the cell (Stelzl et al., 2017).

Furthermore, DNA-based nanoscale materials have attracted a large amount of attention in recent years. Particularly, the programmable design of DNA origami has found promising application in many fields, such as cancer therapy (Rajagopalan and Yakhmi, 2017). Hence, the knowledge of how DNA mechanics operates from atomistic level (nanometer) to macromolecule level (micrometer) will undoubtedly advance our understanding of the machinery of living organisms and grant us more control in designing nanomaterials and nanomachines based on DNA.

As an indispensable tool in DNA study, molecular modeling has advanced considerably in the past two decades. Developers continuously improve the extensively used all-atom (AA) force field models. Many AA force field deficiencies were exposed and subsequently corrected after microsecond-long AA simulations become available (Hart et al., 2012; Galindo-Murillo et al., 2016). With extensive simulations, we have gained atomic-level insights on DNA dynamics (Lavery et al., 2009), DNA flexibility (Minhas et al., 2020), and other physical properties of DNA.

Despite the success of AA DNA models in studying DNA dynamics, flexibility, and binding properties, many important questions, such as nucleosome organization within chromatin that package DNA in the eukaryotic cell nucleus, are still out of their reach. With recent advances in multi-microsecond AA simulations, partial DNA unwrapping from the histone core of the nucleosome can be characterized in detail (Shaytan et al., 2016). However, other important relative motion between DNA and the histone proteins of the histone octamer (HO) that wraps DNA in nucleosomes (Kono and Ishida, 2020), such as sliding, is still beyond the capability of AA-MD. Lowering the model's resolution by merging groups of highly correlated atoms to individual interaction sites is an effective way to study these features. These low-resolution models are usually called coarse-grained (CG) models. In such models, the fluctuations of unimportant degrees of freedom are implicit. Furthermore, with a low number of degrees of freedom, we effectively deal with a smoother free energy surface of the molecular system. Consequently, CG models speed up the simulation in two ways–there are fewer interacting particles in the simulation system–and faster dynamics due to the smoother free energy surface. Within CG models, we can observe molecular dynamics at a large temporal and spatial scale using widely available computational resources, such as workstations and freely accessible simulation software packages.

The development of CG models is not always straightforward, and it remained something of an art rather than rigorous science for a long time. For simple molecular systems, such as liquids of small molecules, the CG model is usually straightforward with a small number of parameters. It is convenient to tune these parameters to reproduce correct macroscopic quantities, such as density and/or surface tension. It is widely known as “top-down” modeling. As the need for a complex model arises, it is usually challenging, if not impossible, to complete the modeling with only top-down approaches as the number of parameters in the model could be exceedingly large. There is not enough information for all parameters to be determined with high confidence.

Another CG modeling approach relies on the lower level, higher resolution models, commonly referred to as fine-grained (FG) models. These so-called “bottom-up” methods achieve the desired modeling by averaging out the unimportant degrees of freedom. For instance, water molecules are usually implicit in CG modeling of proteins, while their effects on, for example, protein conformation is implicitly included in the effective Hamiltonian of the CG model. Therefore, the central task of bottom-up CG modeling is to derive all parameters of the target CG model with the information presented by an underlying fine-grained model, usually an AA model.

**Figure 1:**
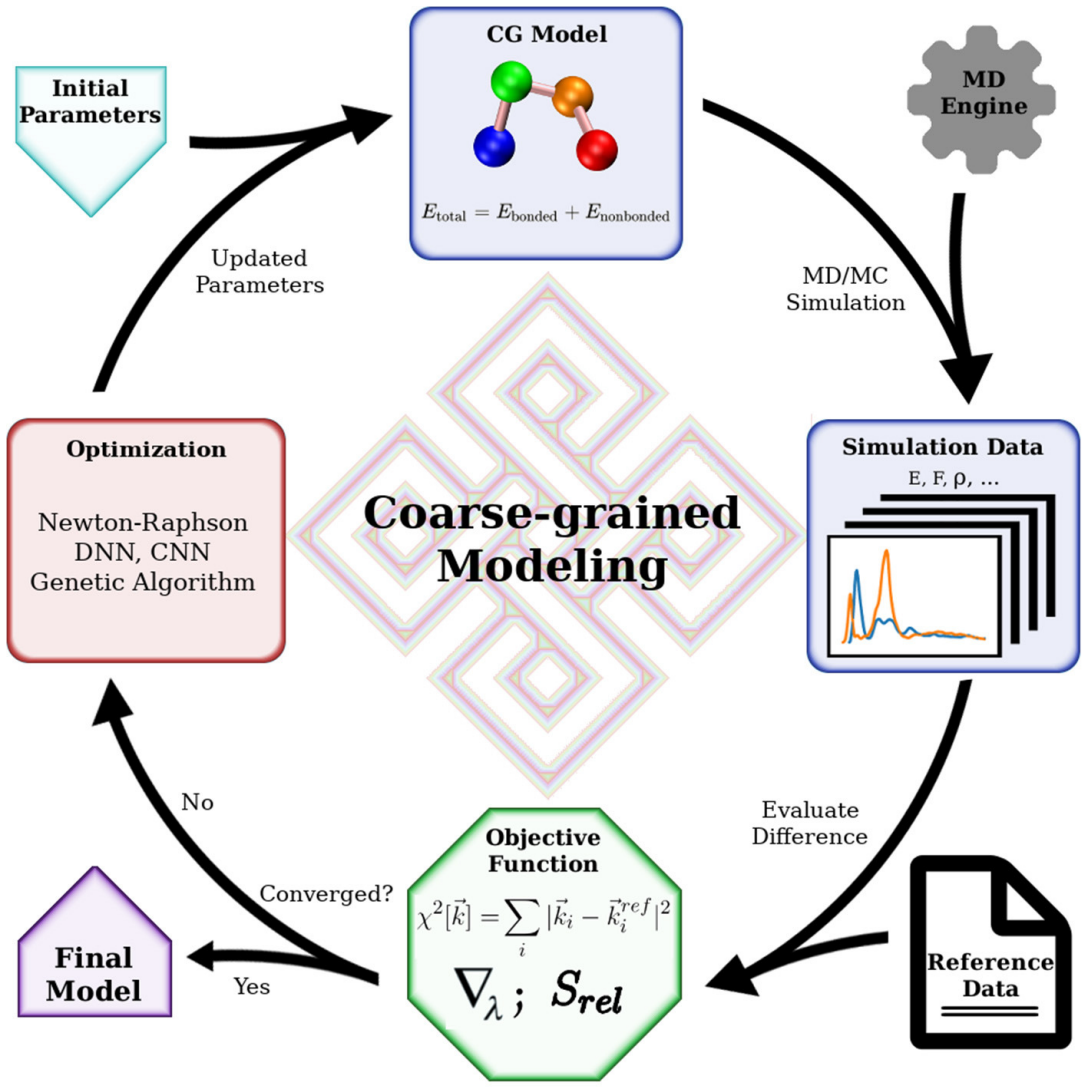
General workflow of coarse-grained modeling. Essential tools for coarse-grained modeling include a software that performs simulation, an objective function to evaluate simulation data and an optimization algorithm to improve the CG model. The final model, which will reproduce the reference data, is obtained when the objective function is satisfied. When the reference data is extracted from fine-grained model simulations, this flow chart represents a general bottom-up coarse-graining algorithm. When the reference data is macroscopic quantities obtained in experiments, this flow chart indicates a top-down coarse-graining procedure. Hybrid coarse-grained modeling is achieved with reference data from both lower-level models and experiments, with a corresponding objective function.

Mathematically, bottom-up coarse-graining strives to project a higher dimensional Hamiltonian to a lower-dimensional space, with the requirement that a certain set of properties is maintained. This set of properties is defined by carefully choosing an objective function that determines the properties of interest and their relative weights. Practitioners have a number of algorithms based on statistical mechanics tackling this projection with various objective functions. Some approaches try to preserve interaction forces by averaging out the fast degrees of freedom by integration (Noid et al., 2008). Some minimize the information loss in the coarse-graining process, characterized by a relative entropy (Shell, 2008, 2016; Chaimovich and Shell, 2011). Others aim to reproduce structural features of the molecular system represented by the radial distribution functions (RDF) (Lyubartsev and Laaksonen, 1995; Soper, 1996). It is worth noting that the objective function does not necessarily consist of elementary physical quantities such as force or particle correlation. It can be a combination of various quantities with predetermined weights, such as distance distributions (Leonarski et al., 2013), force variations (Zhang et al., 2018; Wang et al., 2019), and entropy loss (Wang and Gomez-Bombarelli, 2019). Even macroscopic quantities can be included in such designed objective function to accommodate a wide range of modeling goals. These modeling efforts with designed objective functions gained more popularity in the past decade as machine learning approaches became common. Nevertheless, choosing or designing a suitable objective function is the most critical part in such cases, as the optimization methods are readily available using machine learning algorithms. This process is something of an art, requiring experience in solving such modeling problems. Generally, all CG modeling efforts can be described by a common framework as depicted in Figure 1. The key elements in the framework are the objective function, the optimization algorithm, and the simulation engine. The simulation engine produces simulation results that are evaluated by the objective function with respect to the targeting reference data, before the trial CG model being subjected to optimization. The modeling is successful when the quality of the CG model is good enough judged by the objective function. In this review, we will focus on bottom-up coarse-graining algorithms originating from statistical mechanics. Readers interested in novel objective functions and modeling with machine learning are referred to the review by Gkeka et al. (2020).

Though mathematically well-defined, the bottom-up coarse-graining process is not straightforward in practice, especially for complex molecular models, such as DNA and proteins. Many aspects of interactions and configurations of DNA could collectively determine one specific property, e.g., bending flexibility. It is challenging to model all these kinds of interactions accurately at the same time. Nevertheless, many coarse-grained DNA models, based on atomistic force fields have been developed, following the bottom-up philosophy. In this review, we review the application of bottom-up coarse-graining methods for studying and understanding DNA properties. We will first introduce bottom-up modeling methods in section 2. Some selected representative bottom-up DNA models will be summarized to give an overview of its recent development. The pros and cons of bottom-up modeling different properties of DNA will be presented. Lastly, we will summarize the current stage of bottom-up DNA models and discuss the future development of bottom-up coarse-graining of DNA. DNA is a highly charged polyelectrolyte. The long-range electrostatic interactions between DNA, small mobile ions (due to salt), and other charged small molecules, as well as biomacromolecules, dominates most, if not all, their physicochemical properties (Bloomfield et al., 2000). Dramatic salt effects and the strong influence of the valence of counterions on DNA physical properties are observed. In some cases, it leads to counter-intuitive behavior such as like-charged attraction between the DNA polyions (Guldbrand et al., 1986; Nordenskiold et al., 2008; Korolev et al., 2010, 2016). Bottom-up approaches represents arguably the most rigorous way of extracting the effective electrostatic potentials between the charged CG sites.

## 2. Bottom-up Coarse-Graining

### 2.1. Theoretical Background

In physical terms, bottom-up coarse-graining is the process of removing unimportant degrees of freedom (DOF) from a detailed high-resolution model and formulation of a simpler model, which contains only essential DOFs. Assume that at the high-resolution level, the system is described by a Hamiltonian (potential energy) *H*(**q**_1_, ⋯ , **q**_**n**_), where {**q**_**i**_; *i* = 1, ⋯ , *n*} are coordinates of particles. The potential energy function typically represents the atomistic force field. However, it can be the potential energy of an already existing CG model, or in the case of *ab initio* modeling, can represent the energy surface obtained from quantum chemical computations. Coarse-graining is described in terms of mapping of FG coordinates (degrees of freedom) {**q**_**i**_; *i* = 1, ⋯ , *n*} to CG coordinates {**Q**_**j**_; *j* = 1, ⋯ , *N*} with *N* ≪ *n*, which is mathematically expressed as mapping functions *M*:

(1) $$\begin{array}{l} Q_{j}=M_{j}(q_{1},\cdots ,q_{n}) \end{array}$$

Generally important DOF, represented by coarse-grained sites, can be chosen in different ways, often based on experience grounded in chemical and physical intuition. Typically one chooses CG sites according to the center-of-masses (COM) of the atom groups forming the CG units, while other choices (such as taking coordinates of specific atoms) are also possible. For instance, one can aim to minimize the information loss due to the mapping operation (Giulini et al., 2020), or choose beads representing collective motions (Zhang et al., 2008). The Hamiltonian *H*(**q_1_**, ⋯ , **q_n_**) defines all properties of the high-resolution system and, through the mapping functions (Equation 1), all properties of the CG system. The task of the bottom-up approach is to define the effective interaction potentials for the CG sites, denoted *H*_*CG*_(**Q_1_**, ⋯ , Q_**N**_), which provides the same properties for the CG system as the properties defined by the FG Hamiltonian *H*(**q_1_**, ⋯ , **q**_**n**_) through the CG mapping. In other words, bottom-up modeling is the practice of solving the inverse problem: to determine the CG interaction potential that reproduces known properties obtained from simulations of the FG system.

The CG Hamiltonian, which satisfies the consistency condition, can be deduced from the FG Hamiltonian by integrating over non-interesting degrees of freedom: (Noid et al., 2008; Lyubartsev et al., 2015)

(2) $$\begin{array}{l} H_{CG}(Q_{1},\cdots ,Q_{N})=-\frac{1}{\beta }\ln\int \prod_{i=1}^{n}dq_{i}\prod_{j=1}^{N}\delta (Q_{j}-M_{j}(q_{1},\cdots ,q_{n}))\\ \;exp(-\beta H(q_{1},\cdots ,q_{n}))+C \end{array}$$

The above coarse-grained Hamiltonian, *H*_*CG*_, is also known as an *N*-body potential of mean force. It provides precisely the same structural properties for the CG model as the FG system mapped by Equation (1) to the CG representation. Thermodynamic properties (average energy, free energies, pressure) can also be reproduced, given the fact that the original and CG Hamiltonians have the same partition function. However, a caveat is that the CG Hamiltonian depends on the thermodynamic conditions (temperature, volume, or density). These dependencies need to be considered while obtaining thermodynamic properties by taking the derivative of the partition function with respect to thermodynamic parameters (Lyubartsev, 2018). Reconstruction of correct dynamics in the CG system is a more challenging task, which leads to a generalized Langevin equation with a memory function (Romiszowski and Yaris, 1991). Approximately, dynamics can be reconstructed within a dissipative particle dynamics approach implementing the Mori-Zvanzig formalism (Eriksson et al., 2008; Hijon et al., 2010) or by normal Langevin dynamics with an appropriately chosen friction constant.

However, modeling a CG system using an *N*-body potential (Equation 2) is practically impossible. In all implementation of bottom-up coarse-graining, one resorts to simpler functions such as additive pair potentials. Hence, the task is reformulated into finding the best possible approximation to the exact Hamiltonian in Equation (2), in the form of additive pair interactions:

(3) $$\begin{array}{l} H_{CG}(Q_{1},\cdots ,Q_{N})\approx \Sigma_{i,j}U_{ij}(R_{ij}) \end{array}$$

where *R*_*ij*_ is the distance between CG sites *i* and *j*.

We note that the use of only pair potentials is not a restriction. Other types of interactions can be included in Equation (3). For example, angle (3-body) or torsion (4-body) potential terms are commonly used for macromolecular CG models. Other forms expressing multi-body interactions can also be included as long as they can be handled efficiently by the simulation software. The task of building a CG force field can be reformulated into finding “as good as possible” an approximation to the exact many-body potential according to Equation (3). For instance, finding the best fit of forces coming from both sides of Equation (3) gives rise to the force-matching method (Ercolessi and Adams, 1994; Izvekov et al., 2004), also known as the Multi-Scale Coarse-Graining (MS-CG) method (Izvekov and Voth, 2005). Another approach that rests on minimizing the entropy difference between FG and CG models corresponds to the relative entropy minimization method (Shell, 2008).

All bottom-up approaches can be approximately divided into two categories: thermodynamics-based coarse-graining aiming at a reproduction of thermodynamic properties (free energies, average forces), and structure-based coarse-graining, aiming at the reproduction of structural properties of the FG system. The theoretical justification of structure-based coarse-graining is the Henderson theorem (Henderson, 1974) that defines a one-to-one relationship between a set of radial distribution functions (RDF) and a set of pair potentials for CG sites. Rudzinski and Noid (2011) later generalized the Henderson theorem to include multiple RDFs between different types of CG sites and intramolecular structural properties such as bond lengths and angles distributions. Below we present several well-developed bottom-up CG modeling methods and discuss the connections among them.

### 2.2. Iterative Boltzmann Inversion

We first discuss the simplest algorithm of bottom-up coarse-graining, which is Iterative Boltzmann Inversion (IBI). This method is usually categorized as structure-based coarse-graining, where the pair potential approximation (Equation 3) is fitted to reproduce various distribution functions obtained in atomistic simulations. Common target distributions include radial distribution functions (RDF), bond length distributions, angle value distributions, etc. The IBI approach is implemented through an iterative algorithm (Soper, 1996; Reith et al., 2003). The pair interaction potential is in each iteration updated with a correction term originating from the mean-field approximation:

(4) $$\begin{array}{l} U_{\alpha \beta }^{i+1}=U_{\alpha \beta }^{i}-k_{B}T\ln\left(\frac{g_{\alpha \beta }^{i}\left(r\right)}{g_{\alpha \beta }^{ref}\left(r\right)}\right) \end{array}$$

The pair potential between site types α and β used in iteration *i* + 1 is obtained by applying the correction term (second term on the right-hand side) to the pair potential in iteration *i*. To determine the correction, the pair RDF, $g_{\alpha \beta }^{i}\left(r\right)$, is obtained through proper sampling with the current interaction potential, $U_{\alpha \beta }^{i}$, and subsequently compared with the reference RDF, $g_{\alpha \beta }^{ref}\left(r\right)$, from an FG simulation. The iterative algorithm is started with a bootstrapping potential, usually, a simple potential of mean force: (Soper, 1996; Reith et al., 2003)

(5) $$\begin{array}{l} U_{\alpha \beta }^{0}\left(r\right)=-k_{B}T\ln\;g_{\alpha \beta }^{ref}\left(r\right) \end{array}$$

The convergence of the effective potential is expected when the correction term approaches zero, which also means (Equation 4) that the CG RDF is nearly equal to the RDF of the reference FG simulations. Thus, the inverse problem is solved, and the effective potential is obtained.

Correction of the potential according to Equation (4) is straightforward to implement. This correction is determined only by the value of the same distribution function, while correlations between different interaction terms are completely neglected. As a result, the IBI approach often faces convergence problem even for relatively simple systems such as ion solutions (Hess et al., 2006), where RDFs between different pairs of anions and cations are strongly correlated with each other. In the practical calculation of CG potentials by RDF inversion, it might be instructive to start the iterative process using the IBI approach. This brings the system RDFs close to the reference values, after which one may switch to other algorithms, which take into account correlations between different interaction terms and provide better convergence when the RDFs are close to the reference functions.

### 2.3. Inverse Monte Carlo

The Inverse Monte Carlo (IMC) method (Lyubartsev and Laaksonen, 1995; Lyubartsev, 2018) (also known as Newton Inversion; Lyubartsev et al., 2010) is a general method to invert ensemble averages, and particularly RDFs, to effective pair potentials. For any multi-component system, it goes through an inverse process and produces as output the effective pair potentials between CG sites, which in direct CG simulations reproduce the same RDFs as those obtained in the detailed FG simulations.

Within the IMC approach, both RDFs and interaction potentials are discretized into two sets of values: histogram of particle-particle distances {*G*_α_; α = 1, ⋯ , *m*}, which after ensemble averaging and normalization to bulk particle density yields RDF, and tabulated pair potentials {*U*_α_; α = 1, ⋯ , *m*}, which are determined for the same set of distances as the RDFs. In standard simulations (direct problem), we have the interaction potential *U* as input, and by running MC or MD simulation, we can evaluate averages {〈*G*_α_〉} and thus obtain the RDF as output. In coarse-graining by IMC, we solve the inverse task: from averages determined in FG simulations $\left(\left\{\langle G_{\alpha }^{ref}\rangle \right\}\right)$ we determine the CG effective potential *U*.

The solution to this non-linear inverse problem can be obtained iteratively by the Newton-Raphson method (hence named the Newton Inversion). Let us determine the Jacobian of the *G*(*U*) dependence by defining its elements:

(6) $$\begin{array}{l} J_{\alpha \gamma }=\frac{\partial \langle G_{\alpha }\rangle }{\partial U_{\gamma }} \end{array}$$

This Jacobian (an *m* × *m* matrix) determines how changes of the potential are related to the changes of the RDF:

(7) $$\begin{array}{l} \Delta \vec{G}=J\Delta \vec{U} \end{array}$$

where we use vector notations for the sets of values of the potential and RDF. Respectively, corrections to the potential, which produce the desired changes of the RDF are determined by the inverse matrix:

(8) $$\begin{array}{l} \Delta \vec{U}=J^{-1}\left(\vec{G}-{\vec{G}}^{ref}\right) \end{array}$$

The Jacobian itself can be computed in Monte Carlo or MD simulations by (Lyubartsev and Laaksonen, 1995):

(9) $$\begin{array}{l} \frac{\partial \langle G_{\alpha }\rangle }{\partial U_{\gamma }}=-\frac{1}{k_{B}T}\left(\langle G_{\alpha }G_{\gamma }\rangle -\langle G_{\alpha }\rangle \langle G_{\gamma }\rangle \right) \end{array}$$

Recall that *G* is discretized RDF and *U* is discretized potential energy, as formulated in IMC and implemented in the MagiC software (Mirzoev et al., 2019). More generally, *G* can be any set of observables, and *U*_γ_ can represent an arbitrary parameter of potential energy. The *G*(*U*) dependence in this general sense can be computed in similar ways as in Equation (9), which has been used in the molecular renormalization group (Savelyev and Papoian, 2009a,b, 2010) and ForceBalance (Wang et al., 2014) methods.

Equations (8) and (9) allow us to solve the inverse problem by an iterative procedure. We start from a trial set of potential. In practical simulations, one can start either from zero or from the pair potentials of mean force and then run an MC simulation, followed by computation of RDFs expressed in terms of 〈*G*_α_〉, as well as the cross-correlation terms according to Equation (9). It is followed by inverting the Jacobian defined by Equation (6) (which solves the corresponding system of linear equations) to obtain corrections to the interaction potential *U*. The procedure is repeated until convergence.

In practical computations, the direct use of Equations (8) and (9) may lead to divergence since the method is based on linear extrapolation (Equation 7) of a generally non-linear relationship. A simple way to regularize the procedure is to iterate by “small steps” to ensure staying in the linear regime, i.e., to multiply the difference of RDF, $\vec{G}-{\vec{G}}^{ref}$, by a scaling factor 0 < λ < 1.

Dealing with long-range electrostatic interactions, which are important in the coarse-graining of highly charged systems such as DNA, requires special considerations. In the first application of IMC to the ionic solution (Lyubartsev and Laaksonen, 1995), the electrostatic part of the interaction was separately treated in the simulations. The non-electrostatic part of the interactions is expected to be of short range. While the electrostatic interaction is considered invariable, the short-range interaction is optimized by the IMC procedure to reproduce the RDF within the specified cut-off distance.

### 2.4. Molecular Renormalization Group

Savelyev and Papoian (2009a,b) have proposed an approach similar to IMC bottom-up coarse-graining inspired by the renormalization group theory. The so-called Molecular Renormalization Group Coarse-Graining (MRG-CG) relies on iteratively updating the trial interaction potential, using a Jacobian identical to Equation (6). The novelty is that the Jacobian is obtained by

(10) $$\begin{array}{l} \frac{\partial \langle G_{\alpha }\rangle }{\partial K_{\gamma }}=-\frac{1}{k_{B}T}\left[\langle G_{\alpha }\frac{\partial U}{\partial K_{\gamma }}\rangle -\langle G_{\alpha }\rangle \langle \frac{\partial U}{\partial K_{\gamma }}\rangle \right] \end{array}$$

where *K*_γ_ represents an arbitrary parameter of the potential U. It is apparent that when the Hamiltonian takes the form $U=\underset{\alpha }{\sum }K_{\alpha }G_{\alpha }$, Equation (10) becomes Equation (9). Therefore, MRG-CG is a generalization of IMC.

In the original implementation of IMC (Lyubartsev and Laaksonen, 1995), tabulated potentials were used as the default functional form of the coarse-grained potential, as can be expressed as a collection of delta-functions:

(11) $$\begin{array}{l} U_{CG}=\overset{m}{\underset{i}{\sum }}K_{i}\delta_{i}\left(\text{r}-{\text{r}}_{i}\right) \end{array}$$

where *i* goes through all *m* entries of the tabulated potential. In other words, the CG Hamiltonian is expanded on a delta-function basis set, usually with a large number of parameters {*K*_*i*_}. With the generalization introduced by MRG-CG, one can choose a convenient basis set with much fewer parameters for expanding the Hamiltonian. High efficiency is expected if the chosen basis set is optimal.

### 2.5. Relative Entropy Minimization

In this approach, a quantity called the relative entropy based on information theory was proposed to be used as a minimization objective in bottom-up coarse-graining. The relative entropy is defined as (Chaimovich and Shell, 2011)

(12) $$\begin{array}{l} \;S_{rel}=\sum_{i}{\mathbb{P}}_{FG}(i)\ln\frac{{\mathbb{P}}_{FG}(i)}{{\mathbb{P}}_{CG}(M(i))}+{\langle S_{map}\rangle }_{FG}\\ S_{map}(I)=\ln\sum_{i}\delta_{I,M(i)} \end{array}$$

where the summation goes over all configurations {*i*} in FG ensemble, ℙ(*i*) is the probability of configuration *i* in FG ensemble, *FG* and *CG* denote FG reference quantities and CG quantities respectively. Note that the mapping entropy *S*_*map*_ is not dependent on the CG effective potential. It represents the entropy loss of mapping a set of FG microstates {*i*} into one single CG microstate *I*. Under the canonical ensemble, we have the following expression for relative entropy after substituting configurational probabilities:

(13) $$\begin{array}{l} S_{rel}=\beta {\langle U_{CG}-U_{FG}\rangle }_{FG}-\beta \left(A_{CG}-A_{FG}\right)+{\langle S_{map}\rangle }_{FG} \end{array}$$

Here, *A* = −*k*_*B*_*T* ln *Z* is the Helmholtz free energy, and all averaging is performed under the FG ensemble. Note that the free energy term *A*_*CG*_ is evaluated over the FG ensemble, with the operation of the mapping function and the CG potential function. Hence, the term −β(*A*_*CG*_ − *A*_*FG*_) characterizes the free energy difference over the same FG ensemble, but in representations of respective CG and FG models.

From an information theory point of view, relative entropy quantifies the information loss in the coarse-graining process. A good CG model is expected to have minimal information loss when compared to its FG reference model. Therefore, we minimize the relative entropy *S*_*rel*_ to generate a CG model with respect to the adjustable parameters in CG Hamiltonian.

Minimizing relative entropy with respect to an arbitrary parameter λ of CG Hamiltonian requires

(14) $$\begin{array}{l} \;\frac{\partial S_{rel}}{\partial \lambda }=\beta {\langle \frac{\partial U_{CG}}{\partial \lambda }\rangle }_{FG}-\beta {\langle \frac{\partial U_{CG}}{\partial \lambda }\rangle }_{CG}=0\\ \frac{\partial^{2}S_{rel}}{\partial \lambda^{2}}=\beta {\langle \frac{\partial^{2}U_{CG}}{\partial \lambda^{2}}\rangle }_{FG}-\beta {\langle \frac{\partial^{2}U_{CG}}{\partial \lambda^{2}}\rangle }_{CG}+\beta^{2}{\langle {\left(\frac{\partial U_{CG}}{\partial \lambda }\right)}^{2}\rangle }_{CG}\\ \;-\beta^{2}{\langle \frac{\partial U_{CG}}{\partial \lambda }\rangle }_{CG}^{2}>0 \end{array}$$

Similar to other coarse-graining algorithms, the minimization of relative entropy is achieved through iterations. With a simple update rule (Shell, 2008) as:

(15) $$\begin{array}{l} \lambda^{i+1}=\lambda^{i}-\frac{\partial S_{rel}/\partial \lambda }{\partial^{2}S_{rel}/\partial \lambda^{2}} \end{array}$$

the parameter of Hamiltonian, λ, is updated between iteration *i* and *i* + 1 by the negative ratio of the first derivative to the second derivative of *S*_*rel*_ with respect to λ. Here, the minimization of *S*_*rel*_ is implemented as a Newton-Raphson iterative algorithm. In the case of *U*_*CG*_ being linear in λ, i.e., $U_{CG}\left({\text{Q}}^{N}\right)=\lambda f\left({\text{Q}}^{N}\right)+\cdots \;$, where *f*(**Q**^*N*^) is a function depending on CG coordinates, Equation (15) becomes

(16) $$\begin{array}{l} \lambda^{i+1}=\lambda^{i}-k_{B}T\frac{{\langle f\rangle }_{FG}-{\langle f\rangle }_{CG}}{{\langle f^{2}\rangle }_{CG}-{\langle f\rangle }_{CG}^{2}} \end{array}$$

We may note here that when expanding the CG Hamiltonian with a linear basis set, the correction term in Equation (16) is calculated through the correlation of *f*. It indicates connections to structure-based coarse-graining. We will discuss this further in section 2.8.

### 2.6. Force Matching

Initially proposed by Ercolessi and Adams (1994), the force matching method was later given a solid theoretical basis by Izvekov et al. (Izvekov et al., 2004; Izvekov and Voth, 2005; Noid et al., 2008). The objective in force matching is to minimize the difference in the force as quantified by a functional of the force residual:

(17) $$\begin{array}{l} \chi^{2}[F]=\frac{1}{3N}\langle \sum_{I=1}^{N}|f_{I}(q^{n})-F_{I}(M_{Q}^{N}(q^{n})){|}^{2}\rangle \end{array}$$

In this way, given a CG mapping $M_{\text{Q}}^{N}\left({\text{q}}^{n}\right)$, the forces given by the FG force field, ${\text{f}}_{I}\left({\text{q}}^{n}\right)$, as exerted on atoms forming the CG site *I*, are replaced by the force given by the CG force field, ${\text{F}}_{I}\left({\text{Q}}^{N}\right)$. As in other coarse-graining methods, a pairwise basis set, {ϕ_2_(*R*_*IJ*_)}, is usually adopted to expand the CG force field for efficiency:

(18) $$\begin{array}{l} {\text{F}}_{I}\left({\text{Q}}^{N}\right)=\underset{I\neq J}{\sum }\phi_{2}\left(R_{IJ}\right){ê}_{IJ}=\underset{k}{\sum }c_{k}\underset{I\neq J}{\sum }u_{k}\left(R_{IJ}\right){ê}_{IJ} \end{array}$$

where {*u*_*k*_} is a set of B-spline functions, ê_*IJ*_ is the unit vector pointing from bead *I* to bead *J*. The minimization problem of the force residual functional, with respect to a parameter set {*c*_*k*_}, can be solved in a variational manner. In the further development of the force matching method, regularization (Lu et al., 2010) and an iterative algorithm (Lu et al., 2013) were introduced to improve accuracy in reproducing structure correlations.

When working with pair-wise additive potential, the force matching method can also be accomplished by solving a linear system. Early implementation of the force matching algorithm, employed discrete delta functions to represent forces in the form of (Noid et al., 2007)

(19) $$\begin{array}{l} f\left(r\right)=\overset{N_{d}}{\underset{d}{\sum }}f_{d}\delta_{D}\left(r-r_{d}\right) \end{array}$$

with δ_*D*_(*r*) = 1 when −Δ*r*/2 ≤ *r* < Δ*r*/2, and δ_*D*_(*r*) = 0 otherwise. By minimizing the force residual (Equation 17) with respect to the force table elements *f*_*d*_, a linear equation system is obtained (Noid et al., 2007)

(20) $$\begin{array}{l} \underset{d^{\prime }}{\sum }f_{d^{\prime }}G_{dd^{\prime }}=b_{d} \end{array}$$

where

(21) $$\begin{array}{l} b_{d}={\langle \underset{i}{\sum }\underset{j\neq i}{\sum }\left({\vec{F}}_{i}^{I,AA}\cdot {\vec{u}}_{ij}^{I}\right)\delta_{D}\left(r_{i,j}^{I}-r_{d}\right)\rangle }_{I} \end{array}$$

and

(22) $$\begin{array}{l} G_{dd^{\prime }}={\langle \underset{i}{\sum }\underset{j\neq i}{\sum }\underset{k\neq i,j}{\sum }\left({\vec{u}}_{ij}^{I}\cdot {\vec{u}}_{ik}^{I}\right)\delta_{D}\left(r_{ij}^{I}-r_{d}\right)\delta_{D}\left(r_{ik}^{I}-r_{d^{\prime }}\right)\rangle }_{I} \end{array}$$

The symmetric matrix *G* contains all the information, up to three-body correlation, to connect the table elements of CG forces, *f*_*d*_, and forces in the fine-grained ensemble, *b*_*d*_.

### 2.7. Other Coarse-Graining Approaches

While bottom-up coarse-graining, as described, is a rigorous and self-consistent approach, the accuracy of the bottom-up CG model relies on the quality of the underlying fine-grained model. Deficiency in the all-atom force field could result in incorrect behavior of the derived CG model. For instance, in Maffeo et al. (2014), the CG model of single-stranded DNA obtained by IBI from atomistic simulations could not reproduce experimentally measured radius of gyration. A top-down refinement was subsequently applied to non-bonded interactions to improve the accuracy of the resulting CG model. Such a hybrid bottom-up – top-down approach is useful when fine-grained simulation cannot produce the correct ensemble due to either inaccuracy in the FG model or sampling difficulties. The hybrid approach can also be accomplished by constructing a hybrid objective function before optimization by machine learning algorithms (Leonarski et al., 2013; Zhang et al., 2018; Wang and Gomez-Bombarelli, 2019; Wang et al., 2019; Gkeka et al., 2020). The objective function contains contributions from both the fine-grained simulation and macroscopic measurements. An optimal model should be obtained even though the whole process is not trivial as many hyper parameters are involved in the machine learning algorithm and objective function.

Efforts were also made to derive models in ways similar to the development of classical atomistic force fields. In such practice, a model with a generalized representation of certain atom groups (CG site types) is produced, hoping that these types of CG sites can be used as building blocks in applications of modeling macromolecules. Instead of deducing interaction potential between atom types as in classical force fields, the potential of mean force among these CG site types is calculated through simulations of the moieties constituting the CG sites, using a fine-grained model. The extension of the MARTINI force field to DNA (Uusitalo et al., 2015) follows this modeling philosophy. It is still considered a bottom-up modeling approach since it is based on an atomistic force field model. However it is not a systematic approach, as we described above. Self-consistency is lost in such modeling practices.

While evaluating a CG model's quality, a comprehensive view should be taken to balance various criteria with sound reasons. One should recognize the shortcomings of bottom-up coarse-graining when the resulting CG model fails to reproduce experiments. For such cases, one may seek help from other approaches of deriving the CG model, e.g., by combination with a top-down approach.

### 2.8. Connection Among Bottom-Up Coarse-Graining Methods

The Henderson uniqueness theorem (Henderson, 1974; Rudzinski and Noid, 2011) states that for liquids with only pair-wise interactions, under given temperature and density, the pair-wise potential, which gives rise to a given radial distribution function, is unique up to a constant. As such, a connection is immediately clear among all structure-based coarse-graining methods, including IBI, IMC and MRG-CG. For an inverse modeling problem with a given set of RDFs and intramolecular distributions, all structure-based modeling methods will arrive at the same set of pair-wise potential up to numerical precision, as long as they can produce the potential as their answer. In practice, IBI has an inherent limitation of ignoring all correlations between pair-wise interactions, resulting in IBI being less capable of producing CG potentials with good precision, especially in complex systems with many CG site types. when the IBI approach can provide a satisfactory CG model, it is usually the most efficient one since the evaluation of the RDF produced by a trial potential is much faster than the evaluation of correlations between RDFs required by other methods.

Although relative entropy minimization is formulated from very different principles compared to structure-based coarse-graining, it has been shown that a CG system described by pair-wise potentials obtained by the relative entropy minimization reproduces the RDFs of the FG system (Chaimovich and Shell, 2011). Hence, relative entropy minimization leads to the same result in CG modeling as structure-based methods taking into account the Henderson uniqueness theorem. Indeed, similarities between relative entropy minimization and other modeling methods have been point out by Chaimovich and Shell (2011). The equivalence becomes even more evident from the fact that in the practical implementation of the relative entropy minimization described by Shell (2008), the method involves inversion of the same cross-correlation matrix (Equations 9, 16) as in the IMC method.

The interconnection between the force matching approach and structure-based coarse-graining has been analyzed by Rudzinski and Noid (2011). It was shown that both approaches could be formulated in terms of an information function that discriminates between the ensembles generated by atomistic and CG models. While the relative entropy approach (and thus other structure-based CG methods) minimizes the average of the information function, the force matching method minimizes the average of its squared gradient. It is why force matching usually produces particle distributions different from the FG reference distributions in practice.

Furthermore, it was shown (Noid et al., 2007) that the kernel that describes the effects of three-particle correlations in the force matching method is equivalent to the kernel of the Yvon-Born-Green (YBG) equation, which relates equilibrium particle 2- and 3-body correlation functions to the additive pair-wise Hamiltonian. In later work by Lu et al. (2013), the force matching algorithm based on the YBG equation can be applied iteratively to reproduce the RDF of liquids as done in structure-based coarse-graining. Considering the Henderson uniqueness theorem, we see that the iterative force matching method (Lu et al., 2013) produces the same CG model if it is applied to minimize the RDF difference between the FG and CG models.

We see that, though developed from different theoretical backgrounds, there is a common theme among the discussed bottom-up coarse-graining methods. When a pair-wise additive potential is adopted, all methods reside in the generalized Yvon-Born-Green hierarchy, either directly, or through modifications as in the later development of force matching. More generally, as proposed by Shell (2008) and later elaborated by Rudzinski and Noid (2011), relative entropy is a fundamental quantity in bottom-up coarse-graining, which connects all structure-based methods and force-based methods.

## 3. Coarse-Grained DNA Models

Modeling of DNA, both *in vivo* and *in vitro*, is inherently a multiscale problem, which requires several levels of resolution, from atomistic (and perhaps quantum-chemical) to models describing higher-order structures of DNA in chromatin. Recent years have seen many CG DNA models differing by levels of details developed through both bottom-up and top-down approaches. Here we give a brief description of some models with emphasis on bottom-up models. A summary of these models is provided in Table 1.

**Table 1:** Summary of coarse-grained DNA models.

**DNA models**	**Representation**	**Main features**	**Modeling method**	**Achieved modeling target**
IMC DNA	5 beads per two base pairs	Explicit ion; Stable double helical structure	IMC	Reproduces the bending persistence length of dsDNA; Reproduces multivalent ion-induced DNA aggregation
Fan Bonds Model	1 bead per nucleotide	Double helix is maintained by intra- and inter-strand bonds. Explicit ions	MRG-CG or IMC	Bending persistence length of dsDNA can be reproduced with scaled potential or specific bonding structure
“sugar” model	6 beads per nucleotide	Explicitly modeling the sugar ring conformation with a double-well bond; Explicit Ions	Boltzmann Inversion; Energy Relaxation; Empirical adjustments	Reproduced both A-DNA and B-DNA conformations
3SPN DNA Model	3 beads per nucleotide	Specific potential energy terms for base interactions	Top-down modeling for DNA; Relative entropy minimization for ion	Well reproducing DNA melting curve, shapes and curvature

### 3.1. IMC Model of dsDNA

**Figure 2:**
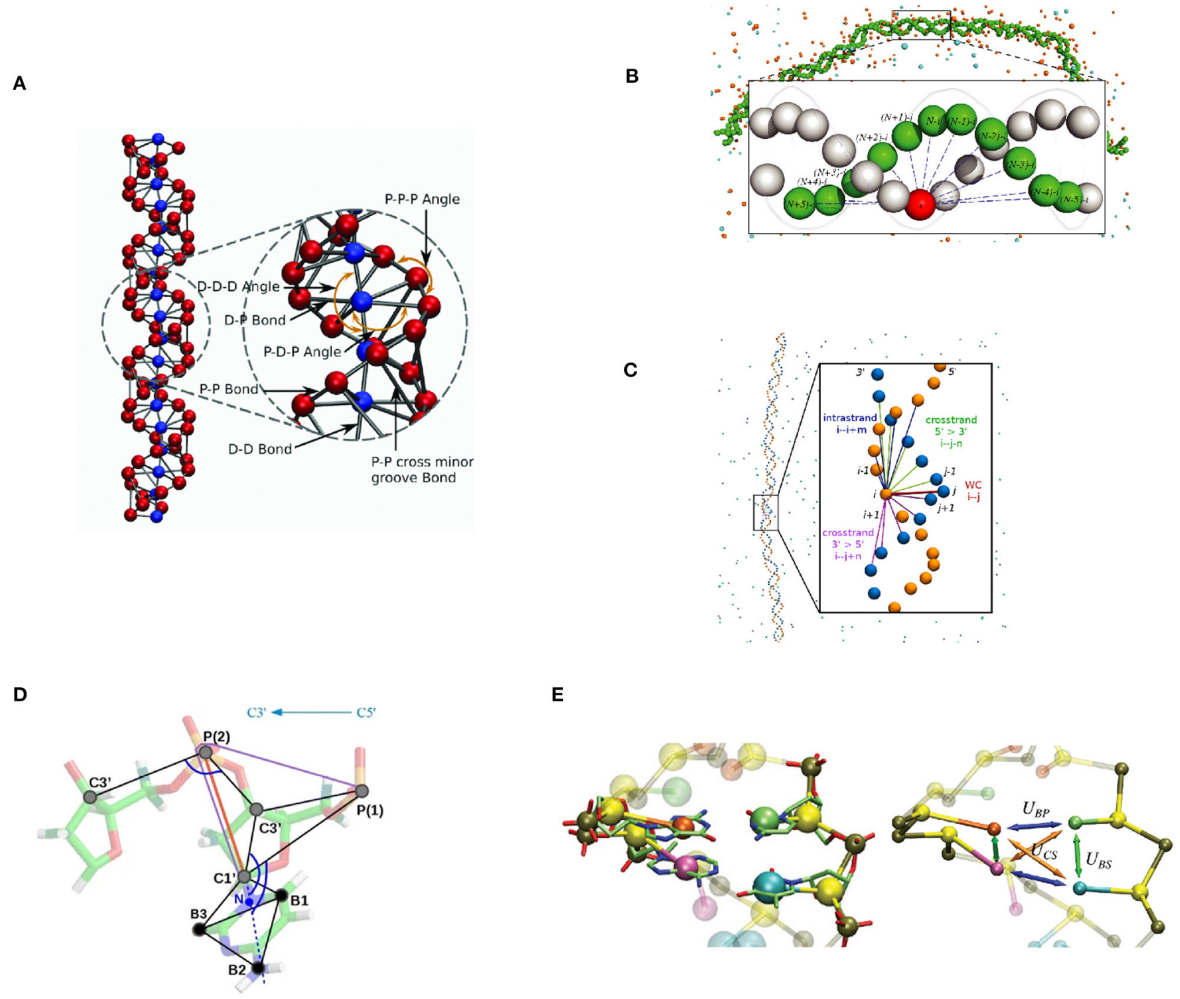
Graphical representation of selected bottom-up coarse-grained DNA models. **(A)** the IMC DNA model (Sun et al., 2019; Minhas et al., 2020). Electrostatic interaction is modeled with explicit ion and phosphate beads (red), in addition to its apparent double-helical structure. Base-pair dynamics is simplified as it is not the primary modeling target. **(B)** Model by Savelyev and Papoian (2010). A double-helical structure is maintained by a series of inter-strand bonds. **(C)** Model by Naômé et al. (2014). Similar to **(B)**. **(D)** The “sugar” DNA model (Kovaleva et al., 2017). Sugar puckering is modeled with a double-well bond potential, so that it is capable of simulating A-DNA and B-DNA transition. **(E)** 3SPN model (Knotts et al., 2007; Sambriski et al., 2009; Hinckley et al., 2013; Freeman et al., 2014). Capable of simulating base-pair dynamics with its detailed Hamiltonian.

We have developed a coarse-grained dsDNA model, focused on the dsDNA double helical shape and a strong emphasis on DNA electrostatic interactions. The first version of this model was proposed by Fan et al. (2013) as a component of a CG nucleosome core particle model. In this model, DNA is represented as a chain of two-base pair units. Each unit contains five CG beads representing two base pairs. Four of the five beads represent phosphate groups with −1*e* charge, denoted “P” beads. The central bead, called the “D” bead, represents the other atoms of these two base pairs, namely four nucleosides, bearing zero charge. Four types of bonds are defined, between “D” bead and “P” beads in the same unit, between adjacent “P” beads on the same strand of DNA, between “D” beads of adjacent units, and lastly, between corresponding “P” beads across the minor groove. These fragments are put together to form a DNA chain with two helices formed by the phosphate groups (Figure 2A).

The total interaction potential consists of bonded and non-bonded interactions:

(23) $$\begin{array}{l} U_{tot}=\underset{bonds}{\sum }U_{bond}\left(r\right)+\underset{angles}{\sum }U_{ang}\left(\phi \right)+\underset{i,j}{\sum }\left(U_{el}\left(r_{ij}\right)+U^{SR}\left(r_{ij}\right)\right) \end{array}$$

where the electrostatic potential is modeled with screened Coulomb potential with ϵ = 78

(24) $$\begin{array}{l} U_{el}\left(r_{ij}\right)=\frac{q_{i}q_{j}}{4\pi \epsilon_{0}\epsilon r_{ij}} \end{array}$$

In the original formulation (Fan et al., 2013), the model was built within a top-down principle, with empirically chosen parameters for intra- and intermolecular interactions. Subsequently, Korolev et al. (2014) developed it into a standalone model of DNA in which intramolecular bonded interactions were parameterized by the IMC approach based on atomistic simulations of DNA with the CHARMM27 force field. For convenience, bonded potentials, *U*_*bond*_(*r*_*ij*_) and *U*_*ang*_(ϕ), obtained by IMC in a tabulated form, were fitted to harmonic potentials and used in such form for subsequent CG simulations. This model has successfully reproduced the persistence length of dsDNA in a wide range of salt concentrations (Korolev et al., 2014).

In a recent effort to implement a full bottom-up model and extend the application range of this model, Sun et al. (2019) have recalculated all interaction potentials for DNA and a number of mono- and multivalent ions by IMC, both bonded and non-bonded. The fine-grained simulation was conducted with four segments of 36-bp dsDNA, described by the CHARMM27 force field. The length of the FG simulations is significantly longer than earlier versions of this model. Interaction potentials involving monovalent ions (Na^+^, K^+^, and Cl^−^) and multivalent ions [Mg(H_2_O)${\;}_{6}^{2+}$ and Co(NH_3_)${\;}_{6}^{3+}$] are derived from the same FG simulations to perform explicit ion simulations on a larger scale with the CG model. Except for the electrostatic potential, all potential terms are derived in tabulated form, as usually done within the IMC algorithm. The model showed its suitability to study multivalent ion-induced DNA aggregation while preserving its accuracy in DNA mechanical properties, i.e., the ion concentration-dependent persistence length (Minhas et al., 2020).

This IMC derived DNA model is designed to work with explicit ions. The model has proven its capabilities in reproducing the mechanical property of dsDNA and phase separation of DNA induced by multivalent ions. The topology of the model preserves the dsDNA's double-helical structure, though it excludes the capability of modeling base pair dynamics, such as bubbling and melting. We will discuss its merits and deficiencies in more detail in the following sections.

A further step of coarse-graining was conducted based on this CG DNA model, producing a mesoscale DNA model (Sun et al., 2019), which showcases the inherent convenience of performing multiscale modeling with bottom-up coarse-graining methods. In this mesoscale model, the dsDNA chain is represented by a chain of spherical beads, each bead representing six base pairs of dsDNA. There are totally three interaction terms in the total potential energy, one bond, one angle and one non-bonded term. With minimal computational resources, simulation of DNA as long as 10 kb was realized. Experimentally observed hexagonal packing in multivalent ion-induced DNA condensates was reproduced in simulations with this mesoscale model. Interestingly, the dynamic process of a single dsDNA chain forming a toroid was described based on simulations within this model.

### 3.2. The Fan Bonds Model

Savelyev and Papoian (2009a,b) have developed a two-bead per base pair model for dsDNA. This model represents each nucleotide with one bead located at its geometric center. In addition to the bonds that connect nucleotide beads on the same strand, the base-pairing and stacking interactions are collectively modeled by a series of inter-strand bonds, which are called “fan” bonds. These interaction terms are designed to maintain the double-helical structure of dsDNA (Figure 2B).

As noted in the theory section above, this Fan Bonds model expands its Hamiltonian over a compact basis set when modeled with the MRG-CG method. The Hamiltonian of this dsDNA model adopts the following form: (Savelyev and Papoian, 2010)

(25) $$\begin{array}{l} \;U_{bond,fan}=\sum_{\alpha =2}^{4}K_{\alpha }{\left(l-l_{0}\right)}^{\alpha }\\ \;U_{ang}=\sum_{\alpha =2}^{4}K_{\alpha }{\left(\theta -\theta_{0}\right)}^{\alpha }\\ U_{ion-DNA;ion-ion}=\sum_{i>j}\left[\frac{A}{r_{ij}^{6;12}}+\sum_{k=1}^{3;5}B^{k}e^{-C^{k}{\left(r_{ij}-R_{ij}^{k}\right)}^{2}}+\frac{q_{i}q_{j}}{4\pi \epsilon_{0}\epsilon r_{ij}}\right] \end{array}$$

Usage of quartic polynomials for bonds and angles and sums of Gaussians for non-bonded interactions significantly reduces the number of parameters. Indeed, the free energy difference between this CG model and the all-atom model is decreased to ~0.5*k*_*B*_*T* in a small number of iterations, which illustrates an efficient algorithm. Finally, the salt concentration-dependent persistence length of dsDNA was reproduced within a scaling factor.

Naômé et al. (2014) have modeled dsDNA with a topology similar to the Fan Bonds model using the IMC approach, while adopting tabulated potentials (Equation 11) to model the system Hamiltonian (Figure 2C). Unlike the model by Savelyev and Papoian (2009a,b), where bonded and non-bonded potentials were derived separately, their model follows a more systematic way of deriving all interaction terms from the same calculation, considering cross-correlation among all interaction terms. The inverse problem was solved in two stages, with IBI and IMC respectively. The final model was obtained after a significantly larger number of iterations (~75 iterations) of than MRG-CG modeling. We clearly see the benefit of having fewer parameters in the MRG-CG method. Various modeling options such as the number of fan bonds and the optimal procedure in solving the inverse problem were explored in their work. The salt concentration-dependent DNA persistence length was well-reproduced with the optimal number of fan bonds, without any scaling factor.

### 3.3. The “sugar” DNA Model

Kovaleva et al. (2017) have formulated the “sugar” DNA model, designed to include the flexibility of the ribose rings. Each nucleotide is represented by six beads – three for the backbone and three for the base (Figure 2D). CG beads are placed on selected atom positions in each nucleotide. The mass of each CG bead is balanced to ensure that the center of mass of the base doesn't move during the mapping operation. The two major conformations of ribose ring, C2′-endo and C3′-endo, are modeled with a double-well potential for P-C1' bond. The transition between these two states corresponds to the transition between C2′-endo and C3′-endo conformations.

Most of the bonded potential functional parameters were derived from atomistic simulations with the AMBER99SB bsc0 force field by Boltzmann inversion, except for the well depth of the aforementioned double-well potential. The depths of two wells of P-C1' potential is set to equal value, as the all-atom sampling is not optimal. In cases where the Boltzmann inversion is not working well, the so-called “relaxation” method is used, where the relevant particle pair is set at a series of distances while relaxing the rest of the system to obtain energy function for this pair. Note that a hybrid approach is adopted in this model to achieve the modeling target. Lastly, using the ion-ion effective potential previously derived by IMC (Lyubartsev and Marčelja, 2002), the “sugar” DNA model successfully modeled the A-DNA and B-DNA states, as well as the transition between the two (Figure 2D).

### 3.4. The 3SPN Model

As an example of a top-down DNA model and hybrid top-down and bottom-up modeling, we discuss the 3-site-per-nucleotide (3SPN) model first developed by Knotts et al. (2007). The first implementation of the 3SPN model (denoted 3SPN.0) was primarily designed to reproduce the melting temperatures of oligonucleotides. Empirically determined relative interaction strengths among non-bonded interaction terms reduce the parameter set to one single interaction energy ϵ, which is subsequently determined with a trial-and-error approach. In the subsequent development, Sambriski et al. (2009) improved the 3SPN model and derived a new version, 3SPN.1. A new solvent-induced attraction term was introduced in this version of the 3SPN model, and other interaction parameters were further tuned to make the model more precise. As a result, DNA mechanics was improved significantly (Figure 2E).

In the subsequent development of the 3SPN model, denoted 3SPN.2 (Hinckley et al., 2013), the authors employed a more detailed interaction Hamiltonian, including a cross-stacking potential to reproduce experimentally determined base interaction energies such as base step energies and base stacking free energies. The resulting model improved the molecular flexibility for both ssDNA and dsDNA. More recently, Freeman et al. (2014) introduced DNA sequence dependence to the 3SPN.2 model. At the same time, additional stability of the helix was implemented using weak dihedral potentials.

Besides developing the 3SPN model by a top-down route, efforts have been made to derive ion-ion, ion-DNA interactions through bottom-up approaches. De Biase et al. (2012) first used a predetermined functional form for ion-ion and ion-DNA interactions, together with 3SPN.1 model. The parameters were derived so that radial distribution functions from all-atom molecular dynamics simulation were satisfactorily reproduced. Subsequently, De Biase et al. (2014) used IMC to derive ion-related potential terms fitted to an empirical functional form. The accuracy of reproducing the RDF was significantly improved in the latter study.

Additionally, the developers of the 3SPN model, Hinckley and de Pablo (2015), developed a transferable coarse-grained ion model for simulations of nucleic acids. Dimethylphosphate (DMP) was adopted as a model molecule for the phosphate group of nucleic acids. Relative entropy minimization was utilized to derive the CG effective potential for ion-ion and ion-phosphate interactions. Ion concentration-dependent persistent length of dsDNA and dsDNA potential of mean force were demonstrated with the new ion model. Although this ionic model only describes ion-ion and ion-phosphate interactions, the authors argued it is a general model, which can be used with other CG models of nucleic acid with explicit charged phosphate sites.

## 4. Discussion

### 4.1. Interactions in CG DNA Models

As straightforward as it is theoretically, modeling interactions of DNA in a CG model is not trivial in practice. Since a few types of interactions are involved, it is difficult, if not impossible, to model all aspects of DNA interactions with a reduced number of DOF accurately. We first consider the practice of modeling the DNA conformation ensemble and discuss modeling interaction between DNA and other molecules or ions later.

An ideal model with general applicability would require fairly accurate modeling of four DNA properties, namely electrostatic interaction, sugar puckering, base-pair stacking, and base-pair hydrogen bonding. Long range electrostatic interactions are crucial to DNA chain conformation and mechanical properties. Sugar puckering is essential in the transition between A-DNA and B-DNA, which in turn contributes to DNA thickness and bending flexibility. For nucleic bases, their stacking and hydrogen bonding interactions are anisotropic and directional due to aromaticity. To our knowledge, there is to date no ideal CG DNA model designed to model all these properties simultaneously. All CG DNA models are compromising and focusing on some particular aspect of the interactions in DNA.

Since most CG models are designed with implicit solvent, various screening methods are usually adopted to model electrostatic interaction, sometimes in conjunction with modifications of charge values (Savelyev and Papoian, 2009b). In such cases, the solvent is treated as a uniform medium without structure. The simplest form of electrostatic interaction potential uses a constant relative permittivity with Coulomb's law:

(26) $$\begin{array}{l} U_{el}=\frac{q_{i}q_{j}}{4\pi \epsilon_{0}\epsilon r_{ij}} \end{array}$$

where ϵ_0_ is the vacuum permittivity, ϵ is the relative permittivity. It is used in the IMC dsDNA model (Korolev et al., 2014; Sun et al., 2019; Minhas et al., 2020), the MRG-CG model (Savelyev and Papoian, 2009a, 2010), the “sugar” model for its ion-ion and ion-DNA interactions (Kovaleva et al., 2017), and a few other models. Some models use the Debye-Huckle potential, generally in the form (Savelyev and Papoian, 2009b; Morriss-Andrews et al., 2010; He et al., 2013; Hinckley et al., 2013; Kovaleva et al., 2017)

(27) $$\begin{array}{l} U_{el}=\frac{q_{i}q_{j}}{4\pi \epsilon_{0}\epsilon r_{ij}}\cdot e^{-r_{ij}/\lambda_{D}} \end{array}$$

where λ_*D*_ is the Debye length of interacting particles. Effectively, interacting particles experience larger permittivity at a longer distance. More complicated forms of distance-dependent permittivity, such as employing a switching function (Kovaleva et al., 2017), are used in some models. Nevertheless, a suitable screened interaction can be engineered to model the electrostatic interaction in CG models where solvent degrees of freedom are missing. However, experience and tweaking might be necessary to obtain an appropriate choice that reproduces a given experimental data-set.

We note here that from the structure-based coarse-graining point of view, electrostatic interaction is part of the force that determines particle correlations alongside short-range interaction, which includes van der Waals attraction, short-range repulsion, and implicit effect of water solvation. With a systematic modeling approach such as IMC, these factors are included in the effective pair interactions as the model's target is to reproduce particle correlation functions. If there was an inaccuracy in the electrostatic interaction, the electrostatic potential's error could be absorbed into the short-range interaction potential and vice versa, such that the final total potential reproduces the target particle correlation functions.

Though sugar puckering is essential to DNA backbone conformation, its modeling is not common in bottom-up CG DNA models, partially due to the inaccuracy in force field parameters and difficulties in the sampling of the C2′-endo and C3′-endo conformations. Additionally, modeling these interactions requires higher resolution, including more CG beads (and DOFs) compared to what is present in most available CG models. The “sugar” DNA model (Kovaleva et al., 2017) explicitly models sugar puckering with a double-well bond potential and a specifically designed bonding structure along the DNA backbone. Additional empirical modifications are needed upon bottom-up modeling to achieve optimal results, manifesting the difficulties mentioned before. Many CG DNA models consider B-DNA only; hence they have not included sugar puckering in their parameter set.

Due to the planar conformation of nucleic bases, interactions originating from them are anisotropic. To realistically represent this anisotropy, there should be a sufficient number of DOF in the CG description. The simplest way is to represent individual nucleic bases with multiple coarse-grained beads. For example, in the “sugar” DNA model, each base is modeled by three CG beads with balanced mass distribution (Kovaleva et al., 2017) such that the base plane is easily defined. In this way, base stacking and hydrogen bonding potentials can be projected onto a few suitable degrees of freedom. Another popular choice is to use anisotropic potentials at interaction sites. The number of interaction sites is minimal, though the number of degrees of freedom is not necessarily small. Gay-Berne potential (Gay and Berne, 1981; Persson, 2012), a generalized Lennard-Jones potential with anisotropy, is frequently adopted to model ellipsoidal CG beads. For instance, ellipsoidal beads are used in the NARES DNA model (He et al., 2013; Liwo et al., 2014; Yin et al., 2015) and used by Li et al. (2016). One should note that using anisotropic beads does not necessarily result in a better model, as seen in Li et al. (2016). No matter through a bottom-up or top-down approach, determining parameters in these models is not a trivial task. Furthermore, anisotropic potentials require more computational time to compute the forces compared to the models based on isotropic distance-dependent potentials. We also note that anisotropic beads may not be necessary for modeling DNA properties intrinsically related to base interactions. For example, the 3SPN model (Knotts et al., 2007) reproduces the dsDNA salt-dependent melting temperature with isotropic beads.

In a simplified representation, DNA base pair interactions can be approximated by bonded fluctuations, as done in the IMC dsDNA model (Korolev et al., 2014; Sun et al., 2019; Minhas et al., 2020) and the G-quadruplex model by Rebič et al. (2015). Though the details of basepair conformation and dynamics are lost, these representations are incredibly efficient in simulation and are easily extended to large molecules. In cases where base pair dynamics is considered secondary, bonded representation could be superior to more detailed base pair models.

### 4.2. Mechanical Properties of DNA

DNA mechanical properties are essential for understanding the DNA behavior in chromatin of the cell nucleus and DNA nanomaterial development. These properties are determined by a combination of DNA intramolecular interactions (backbone rigidity, basepair interactions) and the electrostatic polyelectrolyte nature of DNA. To test the performance of any dsDNA model, the bending flexibility characterized by the bending persistence length is usually the first property tested against known experimental data. In simulations, the bending persistence length, *L*_*p*_, is approximated by an exponential decay of the angular correlation function:

(28) $$\begin{array}{l} \langle {ê}_{i}\cdot {ê}_{i+n}\rangle =\exp\left(\frac{-nI}{L_{p}}\right) \end{array}$$

where **ê**_*i*_ is a unit vector along segment *i*, *I* is the average segment contour length, angle brackets denote ensemble average. Since the importance of the electrostatic interaction for DNA bending flexibility, testing salt concentration-dependent persistence length is a rigorous way to test the performance of a new model. Though, it should be noted that the approximation of an exponential correlation function as above, albeit good for a worm-like chain polymer, is ignoring the DNA sequence effect and intrinsic DNA curvature (Mitchell et al., 2017).

With the IMC dsDNA model, Korolev et al. (2014) tested the salt concentration-dependent persistence length, where the bonded potential is fitted to harmonic function based on IMC inverted potential. The result showed a very good agreement with experimental data. When the bonded potentials were substituted with accurate tabulated IMC inverted potential (Minhas et al., 2020), the bending persistence length still agrees well with experiments. However, when torsion persistence length was tested with this model, the result showed a significantly larger value than experimentally reported data (Korolev et al., 2014). It was explained by reasoning that while ion-dependent DNA bending is determined mostly by long-range electrostatic forces, the torsion flexibility does not depend much on electrostatics. It is determined mostly by basepair twisting, which is relatively short range. Since the IMC dsDNA model simplifies basepair movement to bonded fluctuations, it is challenging to reproduce DNA twisting satisfactorily.

In the two-bead per basepair models by Savelyev and Papoian (2010) and Naômé et al. (2014), the long-range interactions are implemented with explicit ions similarly to the works by Korolev et al. (2014) and Minhas et al. (2020) while the bonding of CG sites along DNA was different. These models show that persistence length is sensitive to the bonding structure. With an optimal bonding structure, the salt-dependent persistence length can be reproduced well.

**Figure 3:**
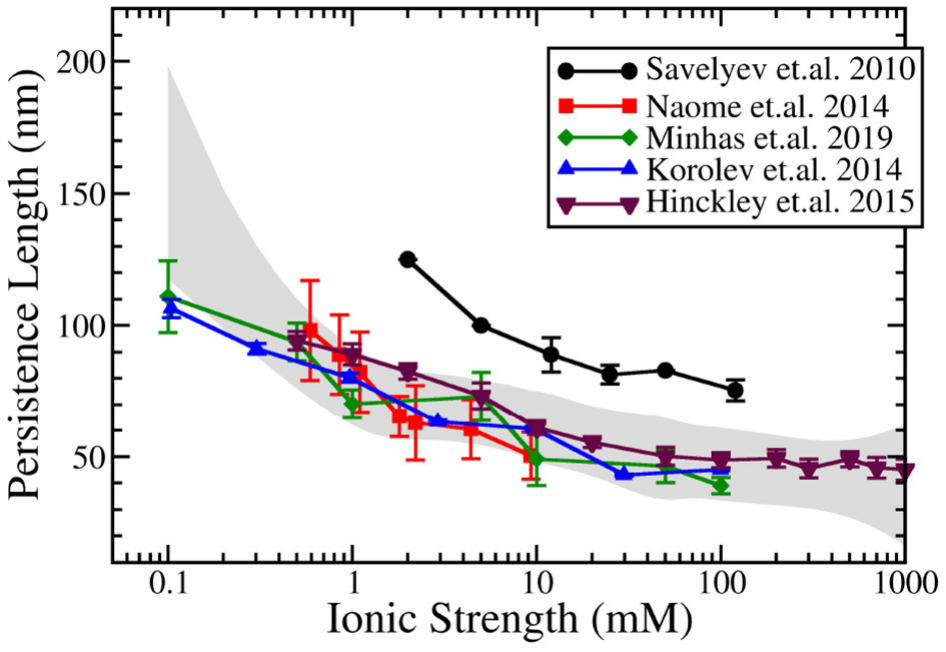
Ionic strength dependent persistence length of dsDNA modeled by coarse-grained DNA models using explicit ions in the simulations. Data from five models are plotted: Savelyev and Papoian (2010), Korolev et al. (2014), Naômé et al. (2014), Hinckley and de Pablo (2015), and Minhas et al. (2020). The gray band corresponds to the spline approximation of the experimental data (Hagerman, 1981; Kam et al., 1981; Manning, 1981; Rizzo and Schellman, 1981; Cairney and Harrington, 1982; Porschke, 1991; Baumann et al., 1997) with a confidence interval of 0.99995.

Figure 3 compares the result of predictions of the dependence of persistence length on salt for these above mentioned bottom-up CG DNA models. Generally, all bottom-up models provide near quantitative agreement with experimental measurements of persistence length over a wide range of salt concentration. The agreement is particularly good at physiological salt. The predictions at salt concentrations below 1 mM display more variation between models and compared to experiments. The results from the IMC model (Minhas et al., 2020) do a good job over a wide range of salt concentrations and are similar to the data obtained by Naômé et al. (2014) and to those of Hinckley and de Pablo (2015). In the latter work, bottom-up derived CG potentials for the ionic interactions were used, while internal DNA interactions were obtained from the empirically parameterized 3SPN model. The persistence length calculations by Savelyev and Papoian (2010), which displayed consistently larger persistence length values, resulted, however,in good agreement with experimental data following uniform rescaling of all CG DNA structural parameters.

If a more detailed CG DNA model is used, it is more challenging to attribute long-range correlation to specific interactions since these are determined collectively by a group of degrees of freedom. For instance, in Morriss-Andrews et al. (2010), the modeled bending persistence length of single-stranded DNA is lower than experiments.

Another interesting mechanical property is force-extension curve of ssDNA. Maffeo et al. (2014) derived a two-bead per nucleotide model with a hybrid approach, i.e., using IBI to derive a primer model, then refined it by fitting to the radius of gyration of ssDNA. The force-extension curve obtained by this model fits better than the other two top-down models to the experimental curve.

### 4.3. DNA Aggregation and Compaction Properties

As a highly-charged polyelectrolyte, DNA has been extensively studied in solution both as a standalone subject and as a component of complexes formed with other molecules. Efforts have been made to model such molecular systems and to get insights from a physical perspective. Besides the bending flexibility discussed in section 4.2, quantities such as radius of gyration (Maffeo et al., 2014), melting temperature (Hinckley et al., 2013), and even knotting probabilities (Rieger and Virnau, 2018) are adopted as modeling targets, especially in top-down models. There are additional studies of DNA-protein complexes with top-down models, such as the NARES model for DNA-protein complex (Yin et al., 2015) and free energy associated with nucleosome unwrapping (Lequieu et al., 2016). Although DNA solution structure can be directly simulated once a bottom-up model is acquired, the result may not always agree with experimental data. For instance, in the ssDNA model designed by Maffeo et al. (2014), the direct result of the CG model derived by IBI significantly underestimates the radius of gyration of ssDNA. Correction to the interaction potential was made afterward to obtain agreement with experimental data.

**Figure 4:**
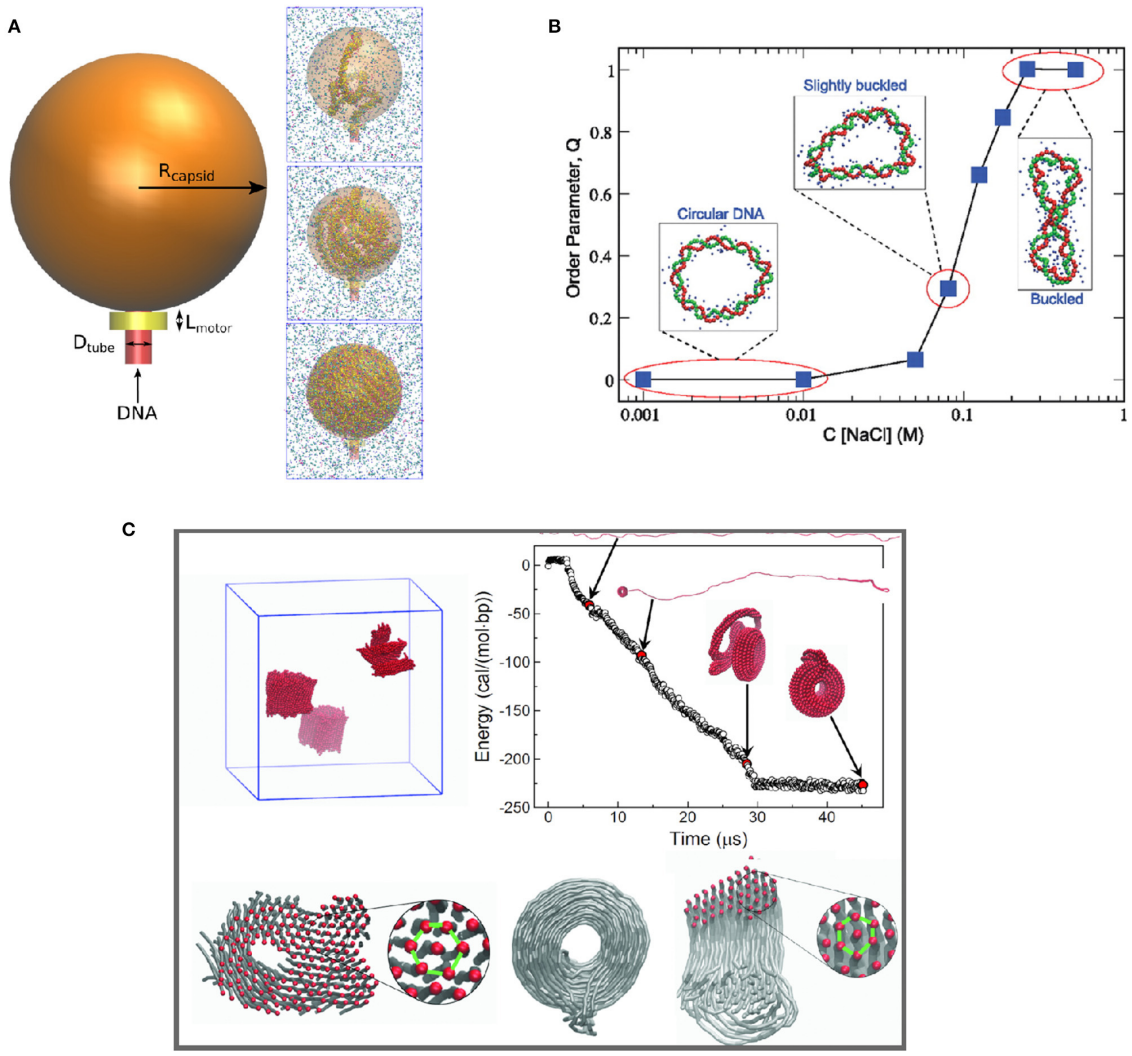
Application of different coarse-grained DNA models illustrating multi-scale phenomena, including **(A)** DNA compaction in virus capsid (Córdoba et al., 2017); **(B)** phase behavior of circular DNA (Savelyev and Papoian, 2010); **(C)** multivalent ion-induced DNA condensation (Sun et al., 2019).

Another application of CG DNA models is to study DNA condensation and phase separation. Positively charged multivalent cations and polyelectrolytes can induce DNA condensation under physiological salt conditions, which is important for understanding DNA compaction in the cell nucleus. Accurate effective potentials between DNA and multivalent ions are crucial to these studies. Córdoba et al. (2017) studied the dependence of DNA packing inside nanometer-sized viral capsids on multivalent cations using the 3SPN.2C CG DNA model incorporating bottom-up effective potentials for ion-phosphate interactions (Figure 4). Multivalent cations such as spermidine and magnesium induce attraction between packaged DNA leading to DNA condensation. At high concentrations of spermidine, the condensation reduced the pressure inside the virus capsid. Savelyev and Papoian (2010) used their CG bottom-up “fan” model to predict the structural phase transitions in torsionally stressed DNA nanocircles due to the presence of salt (see Figure 4B). The model predicted phase transition to a buckled state in the overtwisted DNA nanocircle under physiological salt conditions.

In work by Sun et al. (2019), a fully bottom-up model of dsDNA in the presence of multivalent ions was built based on atomistic simulations with the CHARMM27 force field. Subsequently the model was used to study DNA condensation in the presence of Cobalt(III)-hexammine (CoHex^3+^) ion. The model successfully reproduced the experimentally observed DNA condensation into hexagonally ordered structures (Figure 4C). Furthermore, the developed CG model was used to make one more step in coarse-graining, to obtain a CG DNA model suitable for mesoscale simulation. It resulted in a “super-coarse-grained” DNA model with a simple bead-on-string topology and a single IMC-derived long-range potential between the beads, effectively accounting for the effect of water and ions. The super-CG model of DNA was used to study the formation of toroidal structures formed by long (40k base pairs) DNA in the presence of CoHex^3+^ ions, resulting in excellent agreement with electron microscopy observations of DNA toroids formation. It is worth emphasizing that this model, allowing simulation of DNA on a micrometer length scale, was derived exclusively from atomistic simulations without using any empirical parameters.

## 5. Concluding Remarks

To model large biomolecular systems such as the components of organelles of a living cell (e.g., chromatin in the cell nucleus), an atomistic approach based on all-atom MD simulations is neither computationally feasible nor practically useful since the vast number of DOF makes the analysis complicated. Within the coarse-grained approach, the macromolecular systems can be reduced to a description with effective sites, representing many atoms, which reduces the computational demand and simplifies the analysis by focusing on those DOFs that are of interest for a specific problem at hand. To obtain the effective potentials describing the interactions between the CG sites, we generally have two possibilities, either a top-down approach that fits the potentials to some available experimental data or the bottom-up approach discussed in this review. We have presented an overview of the approach of bottom-up CG computer simulations of DNA, which enables the modeling of multiscale DNA structure, dynamics, and interactions with various bio-macromolecules. As discussed thoroughly here, the available methods are in principle equivalent. They enable rigorous extraction of the effective potentials that reproduces the system's behavior in an FG description based on a given AA force field. These potentials are particularly advantageous when dealing with highly charged systems such as DNA. The IMC method e.g., enables rigorous modeling of DNA mechanical properties and aggregation of very large DNA assemblies, with a reduction of the number of particles of several orders of magnitude, but still using effective potentials that implicitly incorporate the effects of the solvent water. Further development of this approach to model packaging of DNA in chromatin and even chromosomes is a highly challenging problem, which though in principle, can be handled within the same methodology.

We foresee that as the methodology and computational capability improve, physics-based models, derived by bottom-up modeling or from other physical principles, will be taking up more important roles toward illustrating biophysical processes relevant to experimental studies. It is even more evident when one considers the development in experimental techniques, such as Cryo-EM and single-molecule experiments. These experimental methods are reaching into finer and finer scales to probe the underlying physics. The gap between physical simulations and wet-lab experiments is diminishing. With the inherent multiscale nature of bottom-up coarse-graining, multiple molecular models spanning a few magnitude of length scale can be generated rigorously. More accurate interactions will be modeled to conduct simulations. With a minimal number of empirical parameters, simulations can provide better insights into molecular characteristics at each scale. Though there is still much development to be expected in bottom-up coarse-graining methods, we believe the connections between microscopic molecular characteristics and experimental observations will improve, leading to a deeper understanding of DNA physical properties at large spatial and temporal scales.

## Author Contributions

All authors listed have made a substantial, direct and intellectual contribution to the work, and approved it for publication.

## Conflict of Interest

The authors declare that the research was conducted in the absence of any commercial or financial relationships that could be construed as a potential conflict of interest.

## References

[B1] Baumann C. G. Smith S. B. Bloomfield V. A. Bustamante C. (1997). Ionic effects on the elasticity of single dna molecules. Proc. Natl. Acad. Sci. U.S.A. 94, 6185–6190. 10.1073/pnas.94.12.6185 9177192 PMC21024

[B2] Bloomfield V. Crothers D. Tinoco I. Killman P. Hearst J. Wemmer D.. (2000). Nucleic Acids: Structure, Properties, and Functions. Sausalito, CA: University Science Books.

[B3] Cairney K. L. Harrington R. E. (1982). Flow birefringence of t7 phage DNA: dependence on salt concentration. Biopolymers 21, 923–934. 10.1002/bip.360210506 7082770

[B4] Chaimovich A. Shell M. S. (2011). Coarse-graining errors and numerical optimization using a relative entropy framework. J. Chem. Phys. 134:094112. 10.1063/1.3557038 21384955

[B5] Córdoba A. Hinckley D. M. Lequieu J. Pablo J. J. (2017). A molecular view of the dynamics of dsDNA packing inside viral capsids in the presence of ions. Biophys. J. 112, 1302–1315. 10.1016/j.bpj.2017.02.015 28402874 PMC5389966

[B6] De Biase P. M. Markosyan S. Noskov S. (2014). Microsecond simulations of dna and ion transport in nanopores with novel ion-ion and ion-nucleotides effective potentials. J. Comput. Chem. 35, 711–721. 10.1002/jcc.23544 24738152 PMC4018453

[B7] De Biase P. M. Solano C. J. F. Markosyan S. Czapla L. Noskov S. Y. (2012). BROMOC-D: Brownian dynamics/Monte-Carlo program suite to study ion and dna permeation in nanopores. J. Chem. Theory Comput. 8, 2540–2551. 10.1021/ct3004244 22798730 PMC3396124

[B8] Ercolessi F. Adams J. B. (1994). Interatomic potentials from first-principles calculations: The force-matching method. Europhys. Lett. 26, 583–588. 10.1209/0295-5075/26/8/005

[B9] Eriksson A. Jacobi M. N. Nyström J. Tunstrøm K. (2008). Using force covariance to derive effective stochastic interactions in dissipative particle dynamics. Phys. Rev. E 77:016707. 10.1103/PhysRevE.77.016707 18351960

[B10] Fan Y. Korolev N. Lyubartsev A. P. Nordenskiold L. (2013). An advanced coarse-grained nucleosome core particle model for computer simulations of nucleosome-nucleosome interactions under varying ionic conditions. PLoS ONE 8:e54228. 10.1371/journal.pone.0054228 23418426 PMC3572162

[B11] Freeman G. S. Hinckley D. M. Lequieu J. P. Whitmer J. K. de Pablo J. J. (2014). Coarse-grained modeling of dna curvature. J. Chem. Phys. 141:165103. 10.1063/1.4897649 25362344

[B12] Galindo-Murillo R. Robertson J. C. Zgarbova M. Sponer J. Otyepka M. Jurecka P.. (2016). Assessing the current state of amber force field modifications for DNA. J. Chem. Theory Comput. 12, 4114–4127. 10.1021/acs.jctc.6b00186 27300587 PMC4980684

[B13] Gay J. G. Berne B. J. (1981). Modification of the overlap potential to mimic a linear site-site potential. J. Chem. Phys. 74, 3316–3319. 10.1063/1.441483

[B14] Giulini M. Menichetti R. Shell M. S. Potestio R. (2020). An information-theory-based approach for optimal model reduction of biomolecules. J. Chem. Theory Comput. 16, 6795–6813. 10.1021/acs.jctc.0c00676 33108737 PMC7659038

[B15] Gkeka P. Stoltz G. Barati Farimani A. Belkacemi Z. Ceriotti M. Chodera J. D.. (2020). Machine learning force fields and coarse-grained variables in molecular dynamics: Application to materials and biological systems. J. Chem. Theory Comput. 16, 4757–4775. 10.1021/acs.jctc.0c00355 32559068 PMC8312194

[B16] Guldbrand L. Nilsson L. G. Nordenskiold L. (1986). A Monte Carlo simulation study of electrostatic forces between hexagonally packed DNA double helices. J. Chem. Phys. 85, 6686–6698. 10.1063/1.451450

[B17] Hagerman P. J. (1981). Investigation of the flexibility of DNA using transient electric birefringence. Biopolymers 20, 1503–1535. 10.1002/bip.1981.360200710 7023566

[B18] Hart K. Foloppe N. Baker C. M. Denning E. J. Nilsson L. MacKerell A. D. (2012). Optimization of the charmm additive force field for DNA: improved treatment of the bi/bii conformational equilibrium. J. Chem. Theory Comput. 8, 348–362. 10.1021/ct200723y 22368531 PMC3285246

[B19] He Y. Maciejczyk M. Ołdziej S. Scheraga H. A. Liwo A. (2013). Mean-field interactions between nucleic-acid-base dipoles can drive the formation of a double helix. Phys. Rev. Lett. 110:098101. 10.1103/PhysRevLett.110.098101 23496746 PMC3627500

[B20] Henderson R. (1974). A uniqueness theorem for fluid pair correlation functions. Phys. Lett. A 49, 197–198. 10.1016/0375-9601(74)90847-0

[B21] Hess B. Holm C. van der Vegt N. (2006). Osmotic coefficients of atomistic nacl (aq) force fields. J. Chem. Phys. 124:164509. 10.1063/1.2185105 16674148

[B22] Hijon C. Espanol P. Vanden-Eijnden E. Delgado-Buscalioni R. (2010). Mori-Zwanzig formalism as a practical computational tool. Faraday Discuss. 144:301–322. 10.1039/B902479B 20158036

[B23] Hinckley D. M. de Pablo J. J. (2015). Coarse-grained ions for nucleic acid modeling. J. Chem. Theory Comput. 11, 5436–5446. 10.1021/acs.jctc.5b00341 26574332

[B24] Hinckley D. M. Freeman G. S. Whitmer J. K. de Pablo J. J. (2013). An experimentally-informed coarse-grained 3-site-per-nucleotide model of DNA: structure, thermodynamics, and dynamics of hybridization. J. Chem. Phys. 139:144903. 10.1063/1.4822042 24116642 PMC3808442

[B25] Izvekov S. Parrinello M. Burnham C. J. Voth G. A. (2004). Effective force fields for condensed phase systems from ab initio molecular dynamics simulation: a new method for force-matching. J. Chem. Phys. 120, 10896–10913. 10.1063/1.1739396 15268120

[B26] Izvekov S. Voth G. A. (2005). A multiscale coarse-graining method for biomolecular systems. J. Phys. Chem. B 109, 2469–2473. 10.1021/jp044629q 16851243

[B27] Kam Z. Borochov N. Eisenberg H. (1981). Dependence of laser light scattering of dna on nacl concentration. Biopolymers 20, 2671–2690. 10.1002/bip.1981.360201213 7034800

[B28] Knotts T. A. Rathore N. Schwartz D. C. de Pablo J. J. (2007). A coarse grain model for dna. J. Chem. Phys. 126:084901. 10.1063/1.2431804 17343470

[B29] Kono H. Ishida H. (2020). Nucleosome unwrapping and unstacking. Curr. Opin. Struct. Biol. 64, 119–125. 10.1016/j.sbi.2020.06.020 32738677

[B30] Korolev N. Luo D. Lyubartsev A. P. Nordenskiold L. (2014). A coarse-grained dna model parameterized from atomistic simulations by inverse monte carlo. Polymers 6, -1675. 10.3390/polym6061655

[B31] Korolev N. Lyubartsev A. P. Nordenskiold L. (2010). Cation-induced polyelectrolyte-polyelectrolyte attraction in solutions of dna and nucleosome core particles. Adv. Colloid Interface Sci. 158, 32–47. 10.1016/j.cis.2009.08.002 19758583

[B32] Korolev N. Nordenskiold L. Lyubartsev A. P. (2016). Multiscale coarse-grained modelling of chromatin components: DNA and the nucleosome. Adv. Colloid Interface Sci. 232, 36–48. 10.1016/j.cis.2016.02.002 26956528

[B33] Kovaleva N. A. Koroleva I. P. Mazo M. A. Zubova E. A. (2017). The sugar coarse-grained dna model. J. Mol. Model. 23, 1–16. 10.1007/s00894-017-3209-z 28185115

[B34] Lavery R. Zakrzewska K. Beveridge D. Bishop T. C. Case D. A. Cheatham T. homas I.. (2009). A systematic molecular dynamics study of nearest-neighbor effects on base pair and base pair step conformations and fluctuations in B-DNA. Nucleic Acids Res. 38, 299–313. 10.1093/nar/gkp834 19850719 PMC2800215

[B35] Leonarski F. Trovato F. Tozzini V. Les A. Trylska J. (2013). Evolutionary algorithm in the optimization of a coarse-grained force field. J. Chem. Theory Comput. 9, 4874–4889. 10.1021/ct4005036 26583407

[B36] Lequieu J. Cordoba A. Schwartz D. C. de Pablo J. J. (2016). Tension-dependent free energies of nucleosome unwrapping. ACS Central Sci. 2, 660–666. 10.1021/acscentsci.6b00201 27725965 PMC5043429

[B37] Li G. Shen H. Zhang D. Li Y. Wang H. (2016). Coarse-grained modeling of nucleic acids using anisotropic gay-berne and electric multipole potentials. J. Chem. Theory Comput. 12, 676–693. 10.1021/acs.jctc.5b00903 26717419

[B38] Liwo A. Baranowski M. Czaplewski C. Golas E. He Y. Jagiela D.. (2014). A unified coarse-grained model of biological macromolecules based on mean-field multipole-multipole interactions. J. Mol. Model. 20:2306. 10.1007/s00894-014-2306-5 25024008 PMC4139597

[B39] Lu L. Dama J. F. Voth G. A. (2013). Fitting coarse-grained distribution functions through an iterative force-matching method. J. Chem. Phys. 139:121906. 10.1063/1.4811667 24089718

[B40] Lu L. Izvekov S. Das A. Andersen H. C. Voth G. A. (2010). Efficient, regularized, and scalable algorithms for multiscale coarse-graining. J. Chem. Theory Comput. 6, 954–965. 10.1021/ct900643r 26613319

[B41] Lyubartsev A. Mirzoev A. Chen L. Laaksonen A. (2010). Systematic coarse-graining of molecular models by the newton inversion method. Faraday Discuss. 144, 43–56. 10.1039/B901511F 20158022

[B42] Lyubartsev A. P. (2018). Inverse Monte Carlo methods, in Coarse-Grained Modeling of Biomolecules, ed G. A. Papoian (Boca Raton, FL: CRC Press; Taylor &Francis Group), 1–26. 10.1201/9781315374284-133267006

[B43] Lyubartsev A. P. Laaksonen A. (1995). Calculation of effective interaction potentials from radial distribution functions: a reverse Monte Carlo approach. Phys. Rev. E 52, 3730–3737. 10.1103/PhysRevE.52.3730 9963851

[B44] Lyubartsev A. P. Marčelja S. (2002). Evaluation of effective ion-ion potentials in aqueous electrolytes. Phys. Rev. E 65:041202. 10.1103/PhysRevE.65.041202 12005811

[B45] Lyubartsev A. P. Naômé A. Vercauteren D. P. Laaksonen A. (2015). Systematic hierarchical coarse-graining with the inverse Monte Carlo method. J. Chem. Phys. 143:243120. 10.1063/1.4934095 26723605

[B46] Maffeo C. Ngo T. T. M. Ha T. Aksimentiev A. (2014). A coarse-grained model of unstructured single-stranded dna derived from atomistic simulation and single-molecule experiment. J. Chem. Theory Comput. 10, 2891–2896. 10.1021/ct500193u 25136266 PMC4132850

[B47] Manning G. S. (1981). A procedure for extracting persistence lengths from light-scattering data on intermediate molecular weight DNA. Biopolymers 20, 1751–1755. 10.1002/bip.1981.360200815

[B48] Minhas V. Sun T. Mirzoev A. Korolev N. Lyubartsev A. P. Nordenskiold L. (2020). Modeling DNA flexibility: comparison of force fields from atomistic to multiscale levels. J. Phys. Chem. B 124, 38–49. 10.1021/acs.jpcb.9b09106 31805230

[B49] Mirzoev A. Nordenskiold L. Lyubartsev A. (2019). Magic v.3: an integrated software package for systematic structure-based coarse-graining. Comput. Phys. Commun. 237, 263–273. 10.1016/j.cpc.2018.11.018

[B50] Mitchell J. S. Glowacki J. Grandchamp A. E. Manning R. S. Maddocks J. H. (2017). Sequence-dependent persistence lengths of DNA. J. Chem. Theory Comput. 13, 1539–1555. 10.1021/acs.jctc.6b00904 28029797

[B51] Morriss-Andrews A. Rottler J. Plotkin S. S. (2010). A systematically coarse-grained model for DNA and its predictions for persistence length, stacking, twist, and chirality. J. Chem. Phys. 132:035105. 10.1063/1.3269994 20095755

[B52] Naômé A. Laaksonen A. Vercauteren D. P. (2014). A solvent-mediated coarse-grained model of dna derived with the systematic newton inversion method. J. Chem. Theory Comput. 10, 3541–3549. 10.1021/ct500222s 26588318

[B53] Noid W. G. Chu J.-W. Ayton G. S. Krishna V. Izvekov S. Voth G. A.. (2008). The multiscale coarse-graining method. I. A rigorous bridge between atomistic and coarse-grained models. J. Chem. Phys. 128:244114. 10.1063/1.2938860 18601324 PMC2671183

[B54] Noid W. G. Chu J.-W. Ayton G. S. Voth G. A. (2007). Multiscale coarse-graining and structural correlations: connections to liquid-state theory. J. Phys. Chem. B, 111, 4116–4127. 10.1021/jp068549t 17394308 PMC2642678

[B55] Nordenskiold L. Korolev N. Lyubartsev A. P. (2008). DNA-DNA Interactions. Hoboken, NJ: John Wiley &Sons, Ltd. 10.1002/9780470286364.ch8

[B56] Persson R. A. X. (2012). Note: modification of the gay-berne potential for improved accuracy and speed. J. Chem. Phys. 136:226101. 10.1063/1.4729745 22713074

[B57] Porschke D. (1991). Persistence length and bending dynamics of DNA from electrooptical measurements at high salt concentrations. Biophys. Chem. 40, 169–179. 10.1016/0301-4622(91)87006-Q 1653052

[B58] Postow L. Crisona N. J. Peter B. J. Hardy C. D. Cozzarelli N. R. (2001). Topological challenges to dna replication: conformations at the fork. Proc. Natl. Acad. Sci. U.S.A. 98, 8219–8226. 10.1073/pnas.111006998 11459956 PMC37424

[B59] Rajagopalan R. Yakhmi J. V. (2017). Chapter 8: Nanotechnological approaches toward cancer chemotherapy, in Nanostructures for Cancer Therapy, Micro and Nano Technologies, eds A. Ficai and A. M. Grumezescu (Amsterdam: Elsevier), 211–240. 10.1016/B978-0-323-46144-3.00008-8

[B60] Rebič M. Mocci F. Laaksonen A. Uličný J. (2015). Multiscale simulations of human telomeric g-quadruplex DNA. J. Phys. Chem. B 119, 105–113. 10.1021/jp5103274 25469629

[B61] Reith D. Putz M. Muller-Plathe F. (2003). Deriving effective mesoscale potentials from atomistic simulations. J. Comput. Chem. 24, 1624–1636. 10.1002/jcc.10307 12926006

[B62] Rieger F. C. Virnau P. (2018). Coarse-grained models of double-stranded DNA based on experimentally determined knotting probabilities. React. Funct. Polymers 131, 243–250. 10.1016/j.reactfunctpolym.2018.08.002

[B63] Rizzo V. Schellman J. (1981). Flow dichroism of t7 DNA as a function of salt concentration. Biopolymers 20, 2143–2163. 10.1002/bip.1981.360201009 7284565

[B64] Romiszowski P. Yaris R. (1991). A dynamic simulation method suppressing uninteresting degrees of freedom. J. Chem. Phys. 94, 6751–6761. 10.1063/1.460726

[B65] Rudzinski J. F. Noid W. G. (2011). Coarse-graining entropy, forces, and structures. J. Chem. Phys. 135:214101. 10.1063/1.3663709 22149773

[B66] Sambriski E. Schwartz D. de Pablo J. (2009). A mesoscale model of dna and its renaturation. Biophys. J. 96, 1675–1690. 10.1016/j.bpj.2008.09.061 19254530 PMC2717267

[B67] Savelyev A. Papoian G. A. (2009a). Molecular renormalization group coarse-graining of electrolyte solutions: application to aqueous NaCl and KCL. J. Phys. Chem. B 113, 7785–7793. 10.1021/jp9005058 19425537

[B68] Savelyev A. Papoian G. A. (2009b). Molecular renormalization group coarse-graining of polymer chains: application to double-stranded DNA. Biophys. J. 96, 4044–4052. 10.1016/j.bpj.2009.02.067 19450476 PMC2712212

[B69] Savelyev A. Papoian G. A. (2010). Chemically accurate coarse graining of double-stranded dna. Proc. Natl. Acad. Sci. U.S.A. 107, 20340–20345. 10.1073/pnas.1001163107 21059937 PMC2996671

[B70] Schiessel H. (2003). The physics of chromatin. J. Phys. Condens. Matter 15, R699–R774. 10.1088/0953-8984/15/19/203 25563698

[B71] Shaytan A. K. Armeev G. A. Goncearenco A. Zhurkin V. B. Landsman D. Panchenko A. R. (2016). Coupling between histone conformations and dna geometry in nucleosomes on a microsecond timescale: atomistic insights into nucleosome functions. J. Mol. Biol. 428, 221–237. 10.1016/j.jmb.2015.12.004 26699921 PMC4738025

[B72] Shell M. S. (2008). The relative entropy is fundamental to multiscale and inverse thermodynamic problems. J. Chem. Phys. 129:144108. 10.1063/1.2992060 19045135

[B73] Shell M. S. (2016). Coarse-Graining With The Relative Entropy. Hoboken, NJ: John Wiley &Sons, Ltd. 10.1002/9781119290971.ch5

[B74] Soper A. (1996). Empirical potential monte carlo simulation of fluid structure. Chem. Phys. 202, 295–306. 10.1016/0301-0104(95)00357-6

[B75] Stelzl L. S. Erlenbach N. Heinz M. Prisner T. F. Hummer G. (2017). Resolving the conformational dynamics of DNA with Ångstrom resolution by pulsed electron-electron double resonance and molecular dynamics. J. Am. Chem. Soc. 139, 11674–11677. 10.1021/jacs.7b05363 28777549

[B76] Sun T. Mirzoev A. Minhas V. Korolev N. Lyubartsev A. P. Nordenskiold L. (2019). A multiscale analysis of DNA phase separation: from atomistic to mesoscale level. Nucleic Acids Res. 47, 5550–5562. 10.1093/nar/gkz377 31106383 PMC6582353

[B77] Uusitalo J. J. Ingolfsson H. I. Akhshi P. Tieleman D. P. Marrink S. J. (2015). Martini coarse-grained force field: extension to DNA. J. Chem. Theory Comput. 11, 3932–3945. 10.1021/acs.jctc.5b00286 26574472

[B78] Wang J. Olsson S. Wehmeyer C. Perez A. Charron N. E. de Fabritiis G.. (2019). Machine learning of coarse-grained molecular dynamics force fields. ACS Central Sci. 5, 755–767. 10.1021/acscentsci.8b00913 31139712 PMC6535777

[B79] Wang L.-P. Martinez T. J. Pande V. S. (2014). Building force fields: an automatic, systematic, and reproducible approach. J. Phys. Chem. Lett. 5, 1885–1891. 10.1021/jz500737m 26273869 PMC9649520

[B80] Wang W. Gomez-Bombarelli R. (2019). Coarse-graining auto-encoders for molecular dynamics. NPJ Comput. Mater. 5:125. 10.1038/s41524-019-0261-5

[B81] Yin Y. Sieradzan A. K. Liwo A. He Y. Scheraga H. A. (2015). Physics-based potentials for coarse-grained modeling of protein-dna interactions. J. Chem. Theory Comput. 11, 1792–1808. 10.1021/ct5009558 26052263 PMC4455907

[B82] Zhang L. Han J. Wang H. Car R. Weinan E. (2018). DeePCG: constructing coarse-grained models via deep neural networks. J. Chem. Phys. 149:034101. 10.1063/1.5027645 30037247

[B83] Zhang Z. Lu L. Noid W. G. Krishna V. Pfaendtner J. Voth G. A. (2008). A systematic methodology for defining coarse-grained sites in large biomolecules. Biophys. J. 95, 5073–5083. 10.1529/biophysj.108.139626 18757560 PMC2586547

